# Supplementary material for: Detailed characterization of neural selectivity in free viewing primates
Source: Nat Commun. 2023 Jun 20;14:3656. doi: 10.1038/s41467-023-38564-9 (PMC10282080; doi:10.1038/s41467-023-38564-9)
Supplement: Supplementary file 1 — Supplementary Information [file 41467_2023_38564_MOESM1_ESM.pdf]

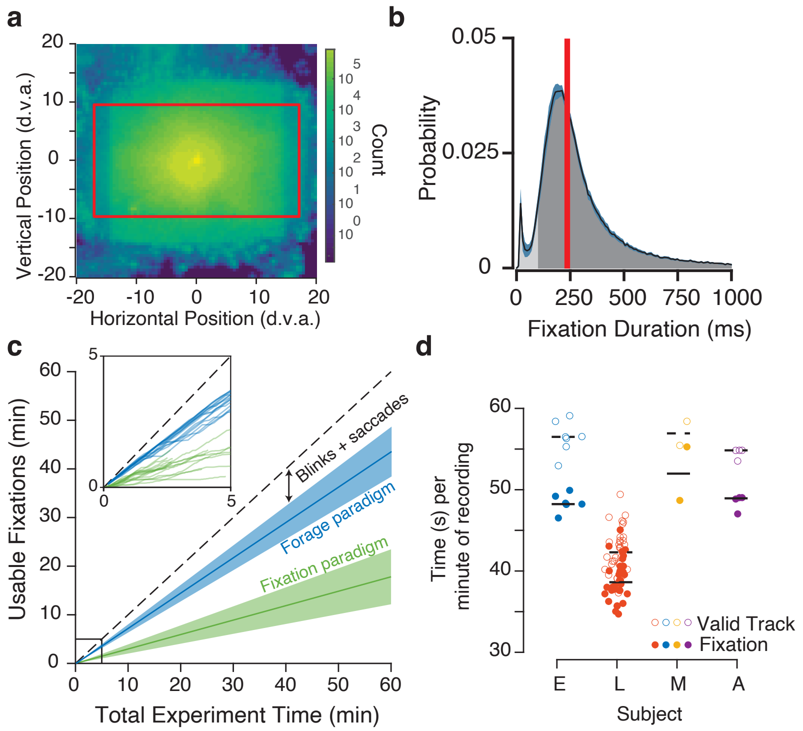

Supplementary Figure 1 | Eye movements during free viewing and usable fixation time. a. Distribution of gaze position for four marmosets during foraging as a heat map. Most time is spent within the central 10 degrees. The circular density at the center is reflective of the annulus from which the forage targets were generated. The red box indicates the size of the projector screen for high-resolution eye tracking sessions. **b.** Distribution of fixation durations during free viewing for 4 marmosets. The dark shaded region indicates the fixations that were included for analyses. Red vertical line indicates the median fixation duration. The peak at short fixation times results from skipping or double-step saccades as have been previously reported in humans (1). **c.** Analysis of the usable fixation time clipping out fixations from free viewing compared to using a fixation paradigm in marmosets. The inset shows measured times for 16 fixation and 45 free viewing sessions in the same marmosets. The shaded plots extrapolate these values for an hour-long session. Solid lines indicate the mean and shaded regions indicate +- 1 standard deviation across 45 sessions. **d.** Fixation time per minute of recording during free viewing for four marmosets [E (7 sessions), L (32 sessions), M (2 sessions), A (4 sessions)]. Open symbols indicate the time with valid eye-tracking (eyes open, track good) and filled symbols are the time spent fixating.

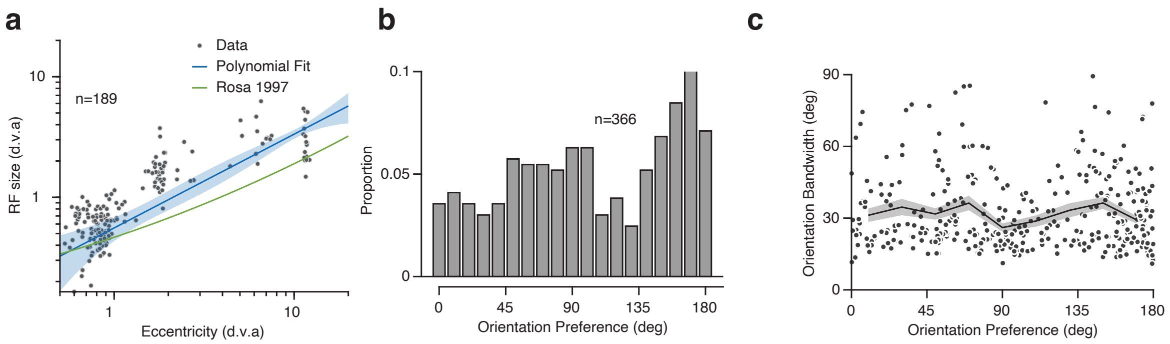

Supplementary Figure 2 | Receptive field size and tuning in V1. a. RF size (square-root of area) as a function of eccentricity (n = 189). Blue line is 2nd-order polynomial fit. Shaded area depicts 95% confidence intervals. Green line is the same polynomial using reported parameters from Rosa et al., 1997. The parameters of the best-fit polynomial were A=0.59 [0.36, 0.81], B = 0.78 [0.39, 1.16], C = 0.00 [-.14, 0.14]. The parameters from Rosa et al., were A=0.76, B=0.49, C = 0.00. **b.** Distribution of preferred orientations measured using parametric fit to the spatial frequency reverse correlation (n=366). The difference in the number of units in panel b and a results from different significance criterion for the grating and spatial noise stimuli. 11 units that had significant tuning to gratings were excluded because they had no orientation tuning (i.e., were only tuned to spatial frequency). **c.** Tuning bandwidth (full-width at half max) as a function of orientation preference, measured from parametric fit (as described in the main text methods). Black line is a running average in 10° bins. The shaded region corresponds to 95% confidence intervals measured with bootstrapping.

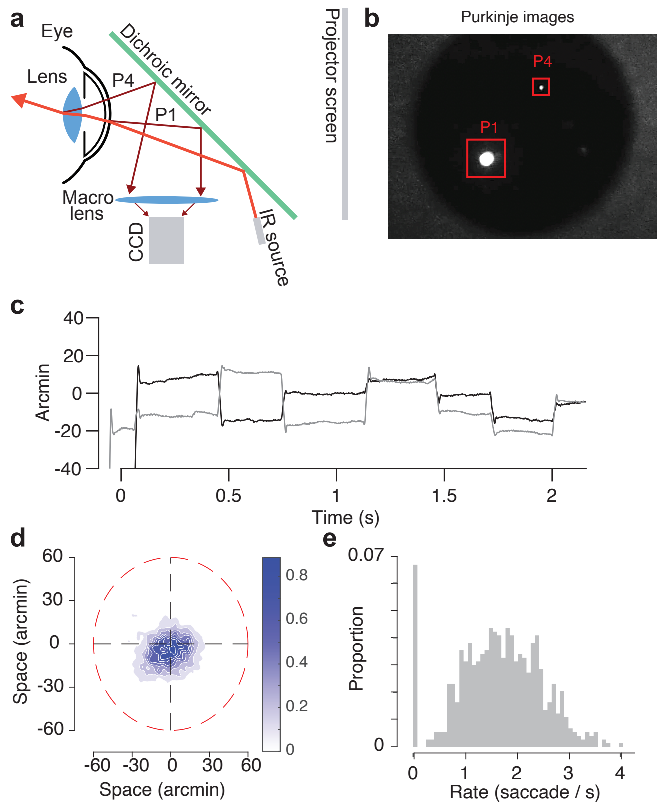

Supplementary Figure 3 | High resolution eye tracking. a. Schematic of the digital Dual Purkinje Imaging (DDPI) system. **b.** Example frame of the pupil with the first and fourth Purkinje images identified. All tracking was performed online using a GPU. **c.** Example horizontal (black) and vertical (gray) gaze position measurements during a fixation trial. This trial was picked because it had many microsaccades within the fixation window. This highlights that fixation often involves a very active process with many eye movements. **d.** Gaze position scatter during fixation. Red dashed line indicates the fixation window that was used online. **e.** Histogram of microsaccade rate during each fixation trial.

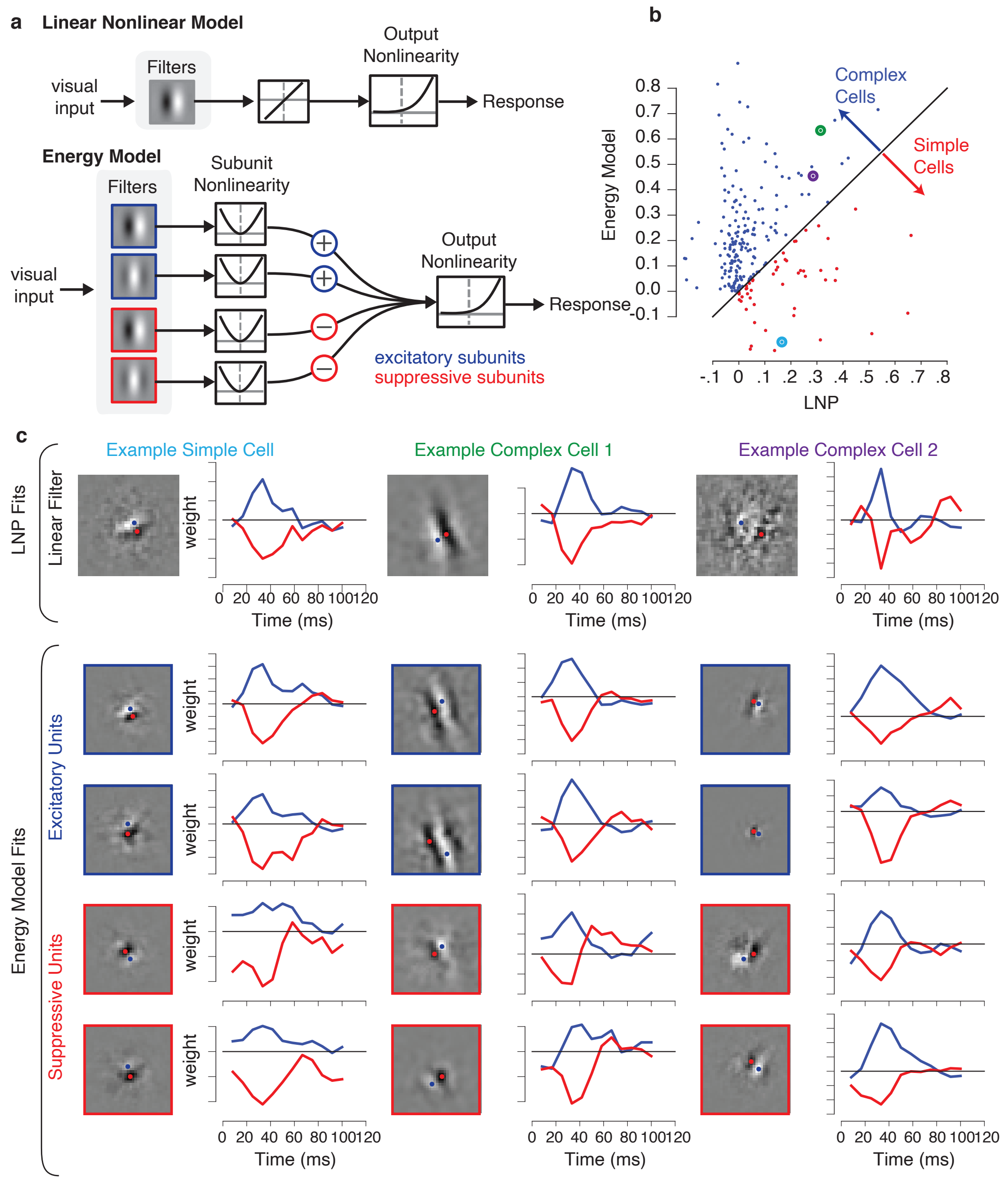

Supplementary Figure 4 | Simple and complex cells. a. Schematics of a simple cell and a complex cell model. Following Vintch et al., 2015, we fit a linear nonlinear Poisson model (LNP) and an Energy Model with quadratic subunits followed by a static nonlinearity and Poisson process. Both models were fit directly to spike counts by minimizing the Poisson loss with L-BFGS. **b.** Log-likelihood (bits/spike) on withheld data for the Energy model and LNP model. Cells to the right of the unity line were classified as simple cells. This classification has been shown to track classic measures of simple and complex cells well (2). Example cells from panel c are highlighted. **c.** Subunits visualized for three example cells shown in panel b. (top row) the weights from the LNP model visualized as the spatial RF at the peak lag and the temporal dynamics of the weights visualized by plotting the spatial locations with the maximum (blue) and minimum (red) values at all time lags. (bottom rows) subunit weights visualized in the same

way as the linear weights above. Each column shows the subunits for the same example cell shown in the top row. Example cell 1 is classified as a simple cell. Although there is spatial structure in its nonlinear subunits, the temporal structure is noisy and the model overfits the training data and cross-validates worse that the LNP model.

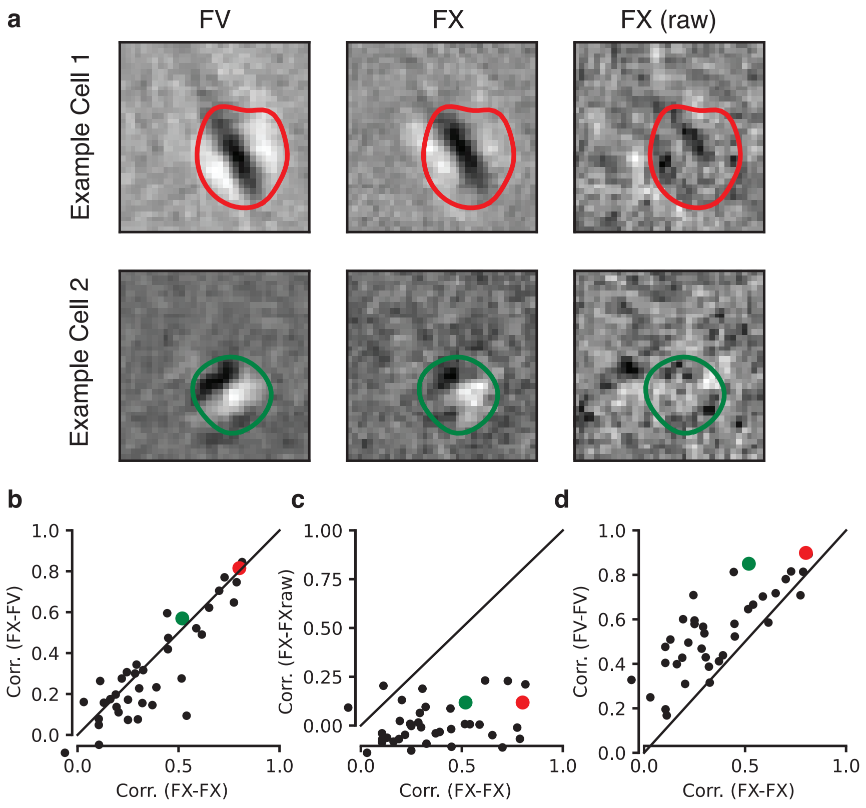

Supplementary Figure 5 | Comparison of the same units during fixation and free viewing. a. Example cells spatial RFs (Spike-Triggered Averages) measured during free-viewing (FV), fixation with correction for measured eye movements (FX) and fixation with no correction (FX (raw)). Contours indicate the region that was correlated to compare across condition. **b.** Scatter plot of RF similarity (Pearson correlation coefficient) within and across conditions. The x-axis is the within-condition correlation plotted for subsets of the fixation data. The y-axis is the RF correlation between fixation and free viewing. The medians are not significantly different (p = 0.31, statistic=820.0, Mann-Whitney U test, two-tailed) **c.** Same as b, except the y axis is the correlation between fixation with correction and fixation without correction. The medians are significantly different (p=8.71×10^{-11}, statistic=1347, Mann-Whitney U test, two-tailed) **d.** same as b,c except the y axis is the within correlation for subsets of free-viewing data. The medians are significantly different (p=0.0009, statistic=402.0, Mann-Whitney U test, two-tailed). The fact that the within correlation is higher for free-viewing suggests we get a more consistent RF in that condition than using fixation.

**Subject**	**Date**	**Units (Total)**	Units (Spatial)	Units (Grating)	**Has RF (Spatial)**	Has RF (Grating)	Duration (Spatial)	**Duration (Gratings)**
**A**	06/01/2022	123	41	50	22	43	149.8	639.6
**E**	07/25/2017	17	15	17	12	16	562.3	1071.7
**E**	07/26/2017	4	3	4	3	3	632.4	1422.6
**E**	07/28/2017	10	9	9	4	7	402.4	972.9
**E**	07/31/2017	34	26	34	10	30	169.2	980.0
**E**	12/24/2018	12	12	12	11	10	914.3	1490.5
**E**	01/07/2019	11	10	10	6	9	906.1	1361.5
**E**	01/11/2019	12	12	12	11	12	1272.8	964.6
**L**	12/02/2019	49	36	36	26	32	133.7	646.7
**L**	12/10/2019	72	25	35	11	31	134.9	648.2
**L**	12/20/2019	53	18	33	11	25	115.3	635.3
**L**	12/23/2019	26	9	9	5	6	128.8	638.2
**L**	12/31/2019	58	19	45	10	43	47.9	649.6
**L**	03/03/2020	68	11	29	5	27	100.1	1279.3
**L**	03/04/2020	65	28	37	16	34	104.2	639.6
**L**	03/06/2020	74	28	35	13	29	78.3	639.6
**M**	06/07/2019	13	11	12	4	12	137	674.1
**M**	06/21/2019	9	9	9	9	8	499.8	580.0

Supplementary Table 1: Sessions contributing to figure 2 and Supplementary figure 2 – coarse spatial mapping / grating tuning. *Subject* indicates the monkey. *Date* indicates the date of the experimental session. *Units (Total)* indicates the total number of units after spike sorting. *Units (Spatial)* and *Units (Grating)* indicate the number of units that met the spike count and waveform criterion during the spatial noise stimulus and grating stimulus, respectively. *Has RF (Spatial)* and *Has RF (Grating)* indicate the number of units that met the significance criterion for the spatial noise and grating stimulus, respectively. *Duration* indicates the duration of stimulus presentation in seconds.

**Subject**	**Date**	**Units (Total)**	Units (Dots)	**Has RF**	**Duration (Dots)**
**E**	1/20/2019	21	21	10	969.2
**M**	2/22/2021	21	21	10	959.0
**M**	4/07/2021	45	45	20	959.0
**M**	4/09/2021	29	29	14	959.0
**M**	5/14/2021	23	23	12	958.8
**M**	5/28/2021	30	30	17	969.0
**M**	7/22/2021	49	49	31	959.0
**M**	8/12/2021	47	47	25	959.0
**M**	8/31/2021	62	62	29	959.0
**M**	9/09/2021	76	76	36	959.0
**M**	9/16/2021	63	63	37	959.0

Supplementary Table 2: Sessions contributing to figure 3 – MT mapping. The header descriptions are the same as in supplementary table 1 except applied to sessions collected with the MT mapping stimulus.

Subject	Date	Units (Total)	Linear RF	Square RF	Duration
**L**	11/19/2019	83	77	73	1081.32
**L**	11/21/2019	67	54	63	793.80
**L**	12/05/2019	36	33	34	415.72
**L**	12/06/2019	39	45	38	501.66
**L**	12/31/2019	58	49	57	475.98
**L**	3/04/2020	65	49	64	517.38
**A**	6/01/2022	123	38	104	701.5
**A**	6/10/2022	150	43	139	724.78

Supplementary Table 3: Sessions contributing to figure 4 – high-resolution V1 RF mapping. The header descriptions are the same as in supplementary table 1.

Supplementary References:

1. Pelz JB, Rothkopf C. Oculomotor behavior in natural and man-made environments. Eye Movements. Elsevier. p. 661–76 (2007).
2. Vintch B, Movshon JA, Simoncelli EP. A convolutional subunit model for neuronal responses in macaque V1. J Neurosci. 35.44. p14829–14841 (2015).
