# Supplementary material for: Detailed characterization of neural selectivity in free viewing primates
Source: Nat Commun. 2023 Jun 20;14:3656. doi: 10.1038/s41467-023-38564-9 (PMC10282080; doi:10.1038/s41467-023-38564-9)
Supplement: Supplementary file 3 — Reporting Summary [file 41467_2023_38564_MOESM3_ESM.pdf]

nature portfolio

Corresponding author(s): Jacob Yates
Last updated by author(s): Nov 15, 2022

Reporting Summary

Nature Portfolio wishes to improve the reproducibility of the work that we publish. This form provides structure for consistency and transparency in reporting. For further information on Nature Portfolio policies, see our Editorial Policies and the Editorial Policy Checklist.

Statistics

For all statistical analyses, confirm that the following items are present in the figure legend, table legend, main text, or Methods section.

n/a	Confirmed	
☐	☒	The exact sample size (n) for each experimental group/condition, given as a discrete number and unit of measurement
☐	☒	A statement on whether measurements were taken from distinct samples or whether the same sample was measured repeatedly
☐	☒	The statistical test(s) used AND whether they are one- or two-sided<br>*Only common tests should be described solely by name; describe more complex techniques in the Methods section.*
☐	☒	A description of all covariates tested
☒	☐	A description of any assumptions or corrections, such as tests of normality and adjustment for multiple comparisons
☐	☒	A full description of the statistical parameters including central tendency (e.g. means) or other basic estimates (e.g. regression coefficient) AND variation (e.g. standard deviation) or associated estimates of uncertainty (e.g. confidence intervals)
☐	☒	For null hypothesis testing, the test statistic (e.g. F, t, r) with confidence intervals, effect sizes, degrees of freedom and P value noted<br>*Give P values as exact values whenever suitable.*
☒	☐	For Bayesian analysis, information on the choice of priors and Markov chain Monte Carlo settings
☒	☐	For hierarchical and complex designs, identification of the appropriate level for tests and full reporting of outcomes
☒	☐	Estimates of effect sizes (e.g. Cohen's d, Pearson's r), indicating how they were calculated

Our web collection on statistics for biologists contains articles on many of the points above.

Software and code

Policy information about availability of computer code

Data collection	All stimulus generation code involved in data collection is provided in a public repository on github: https://github.com/jcbyts/MarmoV5. Preprocessing code is also available on github: https://github.com/jcbyts/MarmoPipe. These links can be found in the methods and in a Code Availability statement as well.
Data analysis	All analyses are available in a public repository on github: https://github.com/VisNeuroLab/yates-beyond-fixation. This link can be found in the code availability statement in the manuscript. MATLAB based analyses were tested on versions R2020b and R2022b. Pytorch analyses provided with the manuscript were tested using Pytorch versions >= 1.7 and <= 1.9.

For manuscripts utilizing custom algorithms or software that are central to the research but not yet described in published literature, software must be made available to editors and reviewers. We strongly encourage code deposition in a community repository (e.g. GitHub). See the Nature Portfolio guidelines for submitting code & software for further information.

Data

Policy information about availability of data

All manuscripts must include a data availability statement. This statement should provide the following information, where applicable:
- Accession codes, unique identifiers, or web links for publicly available datasets
- A description of any restrictions on data availability
- For clinical datasets or third party data, please ensure that the statement adheres to our policy

Full and preprocessed datasets are available from the corresponding author (yates@berkeley.edu) upon reasonable request. An example dataset is available at https://doi.org/10.6084/m9.figshare.22580566

Human research participants

Policy information about studies involving human research participants and Sex and Gender in Research.

Reporting on sex and gender	N/A
Population characteristics	N/A
Recruitment	N/A
Ethics oversight	N/A

Note that full information on the approval of the study protocol must also be provided in the manuscript.

Field-specific reporting

Please select the one below that is the best fit for your research. If you are not sure, read the appropriate sections before making your selection.

☒ Life sciences ☐ Behavioural & social sciences ☐ Ecological, evolutionary & environmental sciences

For a reference copy of the document with all sections, see nature.com/documents/nr-reporting-summary-flat.pdf

Life sciences study design

All studies must disclose on these points even when the disclosure is negative.

Sample size	We obtained the maximum number of neurons available to use in the recording sessions. We analyzed 428 and 621 V1 units for the low and high resolution experiments, respectively, and 466 MT units.
Data exclusions	The cluster outputs from the spike sorting algorithm were excluded if they did not have biologically plausible waveform characteristics, or if they fired fewer than 200 action potentials during a mapping session.
Replication	The method for receptive field recover we describe here is reproduced robustly across multiple experimental sessions, in multiple brain areas, and multiple monkeys. The numbers of each experiment that was recorded are reported in supplemental tables. Experiments supporting figure 2 were replicated 18 times in 3 monkeys. Experiments supporting figure 3 were replicated 11 times in 2 monkeys, and experiments supporting figure 4 were replicated 8 times in 2 monkeys.
Randomization	Experimental stimuli and trial order were randomly generated. In our type of study, all neurons from all experiments are used in a single group so there are no randomly assigned groups.
Blinding	Experimental stimuli and trial order were randomly generated. In this study, statistical models are fit to explore the relationship between a randomly generated stimulus and the firing rate of neurons understudy. Experimenters were blind to the random seeds used in generating stimuli.

Reporting for specific materials, systems and methods

We require information from authors about some types of materials, experimental systems and methods used in many studies. Here, indicate whether each material, system or method listed is relevant to your study. If you are not sure if a list item applies to your research, read the appropriate section before selecting a response.

Materials & experimental systems

n/a	Involved in the study
☒	☐ Antibodies
☒	☐ Eukaryotic cell lines
☒	☐ Palaeontology and archaeology
☐	☒ Animals and other organisms
☒	☐ Clinical data
☒	☐ Dual use research of concern

Methods

n/a	Involved in the study
☒	☐ ChIP-seq
☒	☐ Flow cytometry
☒	☐ MRI-based neuroimaging

Animals and other research organisms

Policy information about studies involving animals; ARRIVE guidelines recommended for reporting animal research, and Sex and Gender in Research

Laboratory animals	This study uses four marmoset monkeys (callithrix jacchus) aged 5, 5, 3, and 7.
Wild animals	No wild animals were used in this study
Reporting on sex	This study uses 1 female and 3 male marmosets. The method worked well in all subjects and no analysis of sex-based effects was performed.
Field-collected samples	No field collected samples were used in this study.
Ethics oversight	All surgical and experimental procedures were approved by the Institutional Animal Care and Use Committee at the University of Rochester in accordance with the US National Institutes of Health guidelines.

Note that full information on the approval of the study protocol must also be provided in the manuscript.
